# Carbohydrate source modulates protein and amino acid digestibility patterns in domestic cats

**DOI:** 10.3389/fvets.2026.1857440

**Published:** 2026-08-14

**Authors:** Woong Ji Lee, Eun Ryul Oh, Jun-Hyeong Seo, Yangseon Kim, Sung Ho Lee, Hyun-Jun Jang

**Affiliations:** 1 Department of Research and Development, Center for Industrialization of Agricultural and Livestock Microorganisms, Jeongeup-si, Republic of Korea; 2 Woogene B&G Co., Ltd., Hwaseong-si, Gyeonggi-do, Republic of Korea

**Keywords:** apparent total tract digestibility, candidate indicator amino acids, efficiency ratio, feline nutrition, food matrix

## Abstract

Domestic cats are obligate carnivores with high dietary protein requirements, yet carbohydrates remain functionally necessary in extruded dry foods. Because carbohydrate matrices may be associated with nutrient accessibility during digestion, this study evaluated whether defined protein- and carbohydrate-source formulations are associated with nutrient and amino acid apparent total tract digestibility (ATTD) in cats. A total of 117 Korean short-haired domestic cats were enrolled and underwent a two-week adaptation period before digestibility evaluation. Twelve diets were formulated to compare six protein sources (chicken, duck, salmon, pollock, beef, and cricket) using rice as a reference carbohydrate source and six carbohydrate sources (rice, corn, potato, tapioca, sweet potato, and wheat) using chicken as a reference protein source. ATTD of nutrients and amino acids was measured, and an efficiency ratio (ER) was calculated as an exploratory descriptive ratio reflecting the relationship between crude protein ATTD and the carbohydrate digestibility denominator. Among protein sources, duck, chicken, and salmon showed the highest protein digestibility (86.36, 85.72, and 83.05%, respectively), whereas beef and cricket showed lower values (74.77 and 74.97%). Among carbohydrate sources, rice and corn showed higher protein digestibility (85.72 and 76.82%, respectively) than wheat, tapioca, potato, and sweet potato (75.23, 74.49, 72.52, and 72.04%, respectively) within the tested chicken-based formulations. Although rice showed the lowest carbohydrate digestibility (7.71%), it yielded the highest ER (11.12), reflecting high crude protein ATTD relative to the carbohydrate digestibility denominator rather than direct evidence of protein utilization efficiency. Seven candidate indicator amino acids (Ala, Ser, Val, Thr, His, Phe, and Ile) were identified based on their strong linear agreement with crude protein ATTD across both source categories. Exploratory group-level regressions showed positive associations between ER and selected candidate amino acid ATTD values, with the strongest relationships observed for Phe (*R*^2^ = 0.89), Ala (*R*^2^ = 0.87), and Ile (*R*^2^ = 0.85). These findings suggest that carbohydrate source may be associated with protein and amino acid ATTD within fixed-matrix feline diet formulations. However, ER should be interpreted as a preliminary, matrix-sensitive descriptive index requiring further validation rather than as a direct measure of protein bioavailability or post-absorptive amino acid utilization.

## 1 Introduction

Pet ownership is increasing worldwide, and with estimates indicate that more than half of all households have cats or dogs. This growth is driven by factors such as increasing incomes, smaller family sizes, an increasing number of people living alone, longer life expectancies, urbanization, and the growing humanization of pets. Consequently, pet food has become one of the fastest-growing product categories globally, with sales increasing to USD$ 125 billion in 2020 (1). With the growing awareness of the critical role nutrition plays in overall well-being, owners are becoming increasingly selective about the food they provide their pets. These dietary choices often mirror the personal values and preferences of the owners and are shaped by various social and cultural influences (2). Thus, sources of macronutrients in pet diets are becoming key factors for both consumers and pet food manufacturers (3).

Protein is an essential component of animal feed. It is necessary for the maintenance of daily body conditions, lactation, growth, weight gain, and reproduction (4). In particular, cats, as obligate carnivores, are metabolically adapted to utilize protein and fat as their main sources of energy. This evolutionary adaptation in energy metabolism compels cats to rely on protein to maintain blood glucose levels, even when dietary protein is limited (5). The significant disparity in protein requirements between cats and omnivores such as dogs highlights this crucial metabolic distinction. For example, kittens and adult cats require approximately 1.5 times and two to three times more protein than omnivore species, respectively (5–7). To date, the Association of American Feed Control Officials (AAFCO) and the European Pet Food Industry Federation (FEDIAF), two key organizations providing guidelines for producing pet food, recommend higher minimum protein levels in cat food, ranging from 25–33.3% on a dry matter basis, compared to 18–25% in dog food. These differences reflect the distinct protein requirements of each species (8, 9).

Carbohydrates are typically absent from a cat’s natural diet, as the meat they consume contains < 5% glycogen on a dry matter basis and no starch. Cats, similar to dogs, have little to no salivary *α*-amylase activity (10), and their pancreatic α-amylase activity is markedly lower than that of dogs and pigs (only 2.3 and 2% of the respective levels in adult animals) (11). In addition, the abundance of glucose transporters in the small intestines of felines is considerably lower than that in canines (12). Consequently, carbohydrates are poorly digested and utilized in the feline small intestine, and carbohydrate-rich diets may induce intestinal inflammation (13, 14). Neither AAFCO nor FEDIAF set minimum carbohydrate requirements for cats. In the AAFCO Cat Food Nutrient Profiles, carbohydrates are not considered essential nutrients, although they are included in metabolizable energy calculations via nitrogen-free extract (NFE) (8). Similarly, the FEDIAF states that there is no minimum requirement for carbohydrates, instead, they state that carbohydrates can serve as an energy source and provide dietary fiber when appropriately processed (9). However, carbohydrate sources, primarily starches, play a critical role in pet food manufacturing. In dry pet-food production, a certain amount of starch is essential to ensure proper processing. The primary function of carbohydrates in extruded dry diets is to provide structural integrity to the kibble; without the binding properties of starch, dry food cannot maintain its shape or structure. Cooked, gelatinized starch acts as the binder that holds the kibble together and prevents crumbling (15). Additionally, interactions between carbohydrates and proteins enhance both the texture and flavor of the final product (16). Although moist foods generally contain a smaller proportion of digestible carbohydrates than dry foods (17, 18), their processing characteristics are also influenced by the carbohydrate content.

Despite these guidelines and the growing demand for pet food, evaluation data on various macronutrient sources in feline diets remain limited. In this study, we supplied various single-protein and -carbohydrate sources to cats and compared their digestibility. Our data will contribute to the development of improved cat food formulations.

## 2 Materials and Methods

### 2.1 Experimental design

Animal study protocols were approved by the Institutional Animal Care and Use Committee of the Center for Industrialization of Agricultural and Livestock Microorganisms (CIALM 2023-02, 2024-04). In total, 117 Korean short-haired cats were initially enrolled in the present study. The cats were provided by the Cat Welfare Association of Southwestern Jeonnam (Animal Shelter). Their ages were determined based on the medical records of the cats at the animal shelter. Their body weights were measured using a veterinary electronic scale. After the preliminary assessments of the cats, cats were initially allocated to the protein-source and carbohydrate-source evaluations and to dietary treatments using a random number generator based on their shelter identification records. During the 2-week adaptation period, feed preference was evaluated under a single-bowl feeding condition by measuring daily refusals. Cats showing persistent refusal or inadequate intake of the assigned diet were excluded before the digestibility evaluation to avoid unreliable nutrient intake estimates. Therefore, the final analyzed population should be interpreted as randomized allocation followed by intake-based eligibility screening rather than as a fully balanced randomized design. The final analyzed population consisted of 117 cats, including 58 cats in the protein-source evaluation and 59 cats in the carbohydrate-source evaluation (Table 1). The final allocation was 8–11 cats per dietary treatment in the protein-source evaluation and 9–10 cats per dietary treatment in the carbohydrate-source evaluation. The individual cat was considered the true experimental unit and one biological replicate. Cats were housed individually in separate cages, corresponding to 58 individual cages for the protein-source evaluation and 59 individual cages for the carbohydrate-source evaluation during the digestibility collection period. Daily intake monitoring and fecal collection were performed individually. The study used an independent-group, non-crossover design, and each cat contributed one final ATTD value per digestibility outcome for statistical analysis; therefore, no repeated-measures model was applied. The protein-source and carbohydrate-source evaluations were not conducted simultaneously; they were conducted sequentially under the same facility, housing, feeding, intake-monitoring, and fecal-collection procedures.

**Table 1:** Characteristics of the feed groups and domestic cats used in the study.

Feed group		No. of cats	Age (month)	Body weight (kg)	Sex ratio (male:female)	Feed preference (%)
Proteins	Chicken	9	14.56 ± 11.16	3.23 ± 1.57	1:1.25	96.17 ± 5.86
	Duck	10	42.60 ± 19.62	5.23 ± 0.88	1:1.20	92.87 ± 9.71
	Salmon	8	44.13 ± 28.40	4.23 ± 1.35	1:1	86.46 ± 11.12
	Pollock	11	48.73 ± 21.36	3.96 ± 1.01	1:0.83	84.64 ± 14.13
	Beef	10	46.60 ± 21.25	3.91 ± 0.86	1:1.5	76.62 ± 11.02
	Cricket	10	31.20 ± 12.90	4.71 ± 0.98	1:1	79.09 ± 20.37
Carbohydrates	Rice	9	14.56 ± 11.16	3.23 ± 1.57	1:1.25	96.17 ± 5.86
	Corn	10	50.00 ± 22.07	4.48 ± 0.87	1:0.67	88.63 ± 7.96
	Potato	10	32.60 ± 31.72	3.73 ± 0.56	1:1	71.64 ± 24.56
	Tapioca	10	23.60 ± 14.29	4.44 ± 1.33	1:1	81.00 ± 20.71
	Sweet potato	10	32.20 ± 17.45	4.71 ± 1.26	1:1.5	71.63 ± 23.77
	Wheat	10	25.20 ± 10.51	4.83 ± 1.24	1:1	74.77 ± 18.78

Feed preference (%) = [(offered − refused)/offered] × 100 during adaptation; this single-bowl measure does not represent a two-bowl preference test.

After the adaptation period, the cats were fed at a fixed rate of 15 g/kg body weight/day. Based on the formulated metabolizable energy values of the experimental diets, this feeding rate provided approximately 49.4–54.5 kcal/kg BW/day across diets, or approximately 52.5 kcal/kg BW/day when calculated using an average dietary ME of 3,500 kcal/kg. Daily refusals were subtracted from the offered amount, and actual intake was used in the ATTD calculations.

### 2.2 Diet formulation

Twelve experimental diets were manufactured by WooGene B&G Co., Ltd. (Republic of Korea) to evaluate six single-protein sources (chicken, duck, salmon, pollock, beef, and crickets; Table 2) and six single-carbohydrate sources (rice, corn, potato, tapioca, sweet potato, and wheat; Table 3). The experimental diets were formulated as dry diets with reference to the AAFCO 2020 Cat Food Nutrient Profiles for adult maintenance. This formulation category was used as the nutritional design target for the short-term digestibility evaluation and was not intended to represent a long-term life-stage feeding claim for all cats in the shelter cohort. The AAFCO 2020 profile was used as the nutritional adequacy reference for formulation targets, including crude protein, crude fat, calcium, phosphorus, and metabolizable energy, whereas the experimental comparison focused on apparent total tract digestibility patterns associated with defined protein and carbohydrate sources. For the protein-source evaluation, rice was selected as the reference carbohydrate source based on previous feline starch-source studies in which rice or brewers rice was evaluated in extruded cat diets (17, 19). For the carbohydrate-source evaluation, chicken was selected as the reference protein source based on previous feline carbohydrate-source diet work using chicken powder as a constant protein ingredient across starch-source diets (19) and prior evaluation of chicken-based protein ingredients intended for dog and cat foods (20). These reference ingredients were selected to reduce formulation complexity and enable comparisons within two predefined dietary matrix contexts. The study was not designed as a full factorial protein × carbohydrate experiment; therefore, the observed responses should be interpreted as matrix-specific outcomes within the tested formulations rather than as intrinsic effects of isolated ingredients alone. Chicken oil was used as the fat source in all diets, with inclusion levels adjusted as needed to meet formulation targets.

**Table 2:** Ingredient information and guaranteed analysis of protein sources for the experiments.

Item	Chicken	Duck	Salmon	Pollock	Beef	Cricket
Ingredient (%)						
Rice	44.4	44.4	44.4	44.4	44.4	38.8
Chicken	30					
Duck		30				
Salmon			30			
Pollock				30		
Beef					30	
Cricket						30
Chicken oil	9.5	9.5	9.5	9.5	9.5	7
Guaranteed analysis (%)						
Moisture	11	11	11	11	11	11
Crude protein	24.5	23	25.5	24	21	24.5
Crude fat	10	10	10	10	10	10
Crude fiber	5	5	5	5	5	5
Crude ash	12	12	12	12	12	12
Calcium (Ca)	1	1	1	1	1	1
Phosphorus (P)	0.8	0.8	0.8	0.8	0.8	0.8
ME [(8); formulated] (kcal/kg)	3,580	3,630	3,550	3,450	3,430	3,590
ME [(8); guaranteed] (kcal/kg)	3,020	3,020	3,020	3,020	3,020	3,020

**Table 3:** Ingredient information and guaranteed analysis of carbohydrate sources for the experiments.

Item	Rice	Corn	Potato	Tapioca	Sweet potato	Wheat
Ingredient (%)						
Chicken	30	33.5	30.5	36	32.5	30
Rice	44.4					
Corn		44.22				
Potato			46.52			
Tapioca				41.52		
Sweet potato					44.52	
Wheat						48.02
Chicken oil	9.5	7	8	7.5	8	7
Guaranteed analysis (%)						
Moisture	11	11	11	11	11	11
Crude protein	24.5	26	26	26	26	26
Crude fat	10	10	10	10	10	10
Crude fiber	5	5	5	5	5	5
Crude ash	12	12	12	12	12	12
Calcium (Ca)	1	1	1	1	1	1
Phosphorus (P)	0.8	0.8	0.8	0.8	0.8	0.8
ME [(8); formulated] (kcal/kg)	3,580	3,380	3,310	3,310	3,290	3,360
ME [(8); guaranteed] (kcal/kg)	3,020	3,020	3,020	3,020	3,020	3,020

### 2.3 Proximate analysis

The chemical compositions of the experimental diets and fecal samples were analyzed using the same standardized procedures. Unless otherwise indicated, the term “sample” in this section refers to either experimental diet or fecal material. Moisture content was analyzed by drying approximately 2 g of finely ground sample before and after drying in a forced-air oven at 135 °C for 2 h and then cooled in a desiccator for 30 min. The crude protein content was analyzed according to AOAC 976.05 using the Kjeldahl method. Briefly, 0.5 g of sample was digested with 12 mL of sulfuric acid and catalyst at 420 °C for 1 h, and the nitrogen content was measured using an automated analyzer (Kjeltec Auto 8,400; FOSS, Sweden). The crude protein content was calculated by multiplying the nitrogen content by a conversion factor of 6.25. The crude fat content was analyzed by ether extraction using an Ankom XT15 extractor (Ankom Technology, United States). Briefly, 0.5 g of sample was dried at 102 °C and then subjected to diethyl ether extraction for 60 min, and the fat percentage was computed from the weight loss of the filter bags. The crude fiber content was analyzed by measuring the weight loss after treating 0.5 g of the sample with 1.25% H_2_SO_4_ and 1.25% NaOH solutions, using a DELTA fiber analyzer (Ankom Technology, United States). Crude ash content was analyzed by pre-incinerating 2 g of sample and then complete combustion using a muffle furnace at 600 °C for 3 h. Gross energy was analyzed using an oxygen bomb calorimeter (PARR 6100; United States) with a 0.5–1 g sample under 35 bar pressure using 99.5% pure oxygen.

### 2.4 Amino acid analysis

Amino acids in the diets were analyzed by ion-exchange chromatography with a post-column derivatization technique using a modified AOAC (19) method. To determine the proportions of the 16 standard amino acids, 0.1 g of the sample was hydrolyzed in 10 mL of 6 N HCl at 110 °C for 24 h in a nitrogen atmosphere. The hydrolysate was then concentrated, reconstituted in 50 mL of 0.2 M sodium citrate buffer, filtered through a 0.20-μm cellulose acetate syringe filter, and analyzed using an amino acid auto-analyzer (Hitachi L-8900, Japan). For the analysis of sulfur-containing amino acids, methionine, and cysteine, the samples were analyzed using a performic acid oxidation technique. Tryptophan analysis was performed using the alkaline hydrolysis coupled with high-performance liquid chromatography (HPLC). The HPLC system consisted of a Waters Isocratic 600 pump, a 486 UV/VIS detector, a CAPCELL PAK C18 column, and a mobile phase of 0.0085 M sodium acetate + methanol (95:5).

### 2.5 Digestibility and efficiency ratio calculations

Apparent total tract digestibility (ATTD) for nutrients and individual amino acids was calculated using the equation: (*A* − *B*)/*A* × 100, where *A* represents the total nutrient content in dietary intake and *B* represents the total nutrient content in fecal output. Daily food refusals were subtracted from the offered amount to determine actual nutrient intake for *A* in the ATTD equation. To describe the relative relationship between crude protein ATTD and carbohydrate ATTD across carbohydrate-source diets, the efficiency ratio (ER) was calculated by dividing the crude protein digestibility percentage by the carbohydrate digestibility percentage. Because ER is derived from two percentage-based digestibility values, it was interpreted as an exploratory descriptive ratio rather than as a direct measure of protein utilization, protein absorption, or post-absorptive amino acid bioavailability.

### 2.6 Statistical modeling

Data processing and statistical analyses were performed using the Pandas, SciPy, and Statsmodels libraries in Python software (version 3.13). To identify candidate amino acids that closely reflected crude protein ATTD patterns, linear regression analysis was performed between crude protein digestibility and individual amino acid digestibility. A coefficient of determination threshold of *R*^2^ > 0.82 was selected *post hoc* based on an apparent separation in the exploratory distribution of *R*^2^ values across all tested amino acids. This threshold was used only to identify amino acids showing strong linear agreement with crude protein ATTD and was not used as a statistical significance criterion. For the treatment-level exploratory regression used to describe the relationship between crude protein ATTD and individual amino acid ATTD, the model AA*i* = *β*0 + *β*1CP*i* + *εi* was used, where AA*i* represents the ATTD of a given amino acid for dietary treatment *i* and CP*i* represents crude protein ATTD for dietary treatment *i*. The relationship between ER and the digestibility of the identified candidate amino acids was assessed using the model AA*i* = *β*0 + *β*1ER*i* + *εi* as an exploratory group-level association. Because these regressions were performed using dietary treatment means, the number of observations was limited to the number of dietary sources. Regression *p*-values and 95% confidence bands were displayed in the corresponding regression visualizations, but residual diagnostics and validation procedures were not used as inferential criteria because of the limited number of treatment-level observations. This analysis was not used to validate ER as a determinant of protein bioavailability or amino acid utilization.

### 2.7 Group comparisons

Differences in digestibility among dietary sources were analyzed separately for the protein-source and carbohydrate-source evaluations using one-way analysis of variance (ANOVA), with dietary source included as the fixed effect and individual cat used as the experimental unit. For group comparisons, the following one-way ANOVA model was used separately within each evaluation context: *Yij* = *μ* + *Si* + *εij*, where *Yij* represents the digestibility response of cat *j* receiving dietary source *i*, *μ* is the overall mean, *Si* is the fixed effect of dietary source, and *εij* is the residual error. When significant differences were detected, Tukey’s honest significant difference (HSD) test was used for post-hoc pairwise comparisons. Sex, age, and body weight were summarized as descriptive animal characteristics in Table 1 but were not included as covariates in the primary ANOVA model. Statistical significance was set at *p* < 0.05.

## 3 Results

### 3.1 Proximate composition of experimental diets

**Figure 1:**
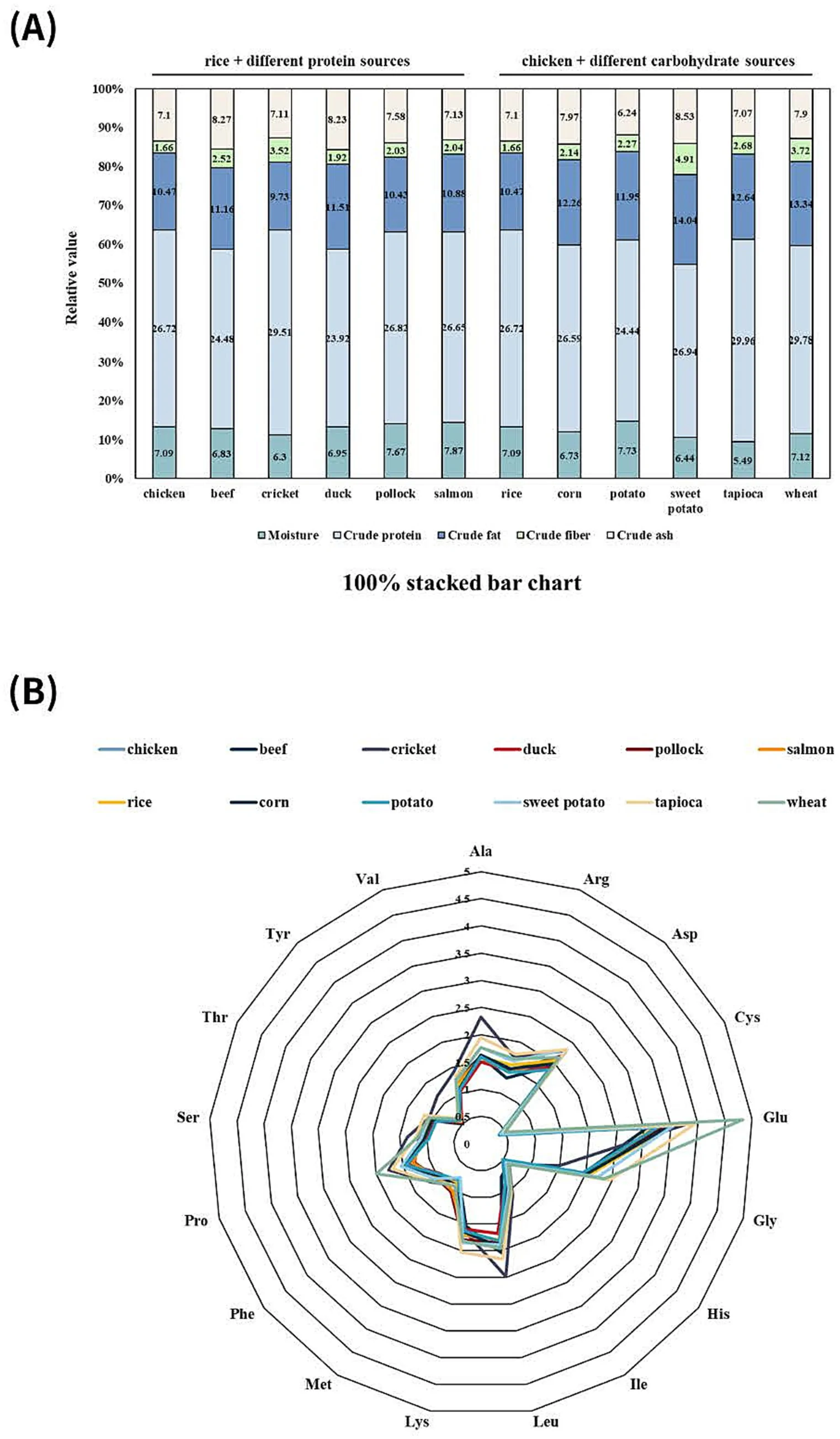
Proximate composition and amino acid profiles of the experimental diets. **(A)** Proximate composition, including moisture, crude protein, crude fat, crude fiber, and crude ash, of the experimental diets prepared using six single-protein sources (chicken, beef, cricket, duck, pollock, and salmon) and six single-carbohydrate sources (corn, potato, sweet potato, tapioca, wheat, and rice). **(B)** Amino acid composition, including the content of 17 individual amino acids, in the formulated experimental diets.

To compare the digestibility of single energy sources, six single-protein source-based feeds, including chicken, beef, crickets, duck, pollock, and salmon, were produced with rice supplied as a common carbohydrate source, and six single-carbohydrate source-based feeds including corn, potato, sweet potato, tapioca, and wheat, were produced with chicken supplied as a common protein source. The proximate composition values were summarized at the diet-formulation level because the analyses were performed using the experimental feed samples rather than animal-level treatment data. Therefore, these values describe the chemical composition of the 12 source-labeled experimental diet formulations. Across all 12 experimental diets, crude protein was 26.88 ± 2.03%, crude fat was 11.57 ± 1.31%, crude fiber was 2.59 ± 0.98%, crude ash was 7.52 ± 0.67%, and moisture was 6.94 ± 0.67% (Figure 1A). To clarify the two predefined evaluation contexts, the same proximate composition data were also summarized separately for the six protein-source diet formulations and the six carbohydrate-source diet formulations. In the protein-source diet formulations, crude protein, crude fat, crude fiber, crude ash, and moisture were 26.35 ± 1.99%, 10.70 ± 0.63%, 2.28 ± 0.67%, 7.57 ± 0.56%, and 7.12 ± 0.57%, respectively. In the carbohydrate-source diet formulations, the corresponding values were 27.41 ± 2.11%, 12.45 ± 1.23%, 2.90 ± 1.21%, 7.47 ± 0.82%, and 6.77 ± 0.76%, respectively. Subsequently, the contents of 17 amino acids including alanine (Ala), arginine (Arg), aspartic acid (Asp), cysteine (Cys), glutamic acid (Glu), glycine (Gly), histidine (His), isoleucine (Ile), leucine (Leu), lysine (Lys), methionine (Met), phenylalanine (Phe), proline (Pro), serine (Ser), threonine (Thr), tyrosine (Tyr), and valine (Val) were analyzed in each feed (Figure 1B).

### 3.2 Nutrient digestibility in the protein-source evaluation

**Figure 2:**
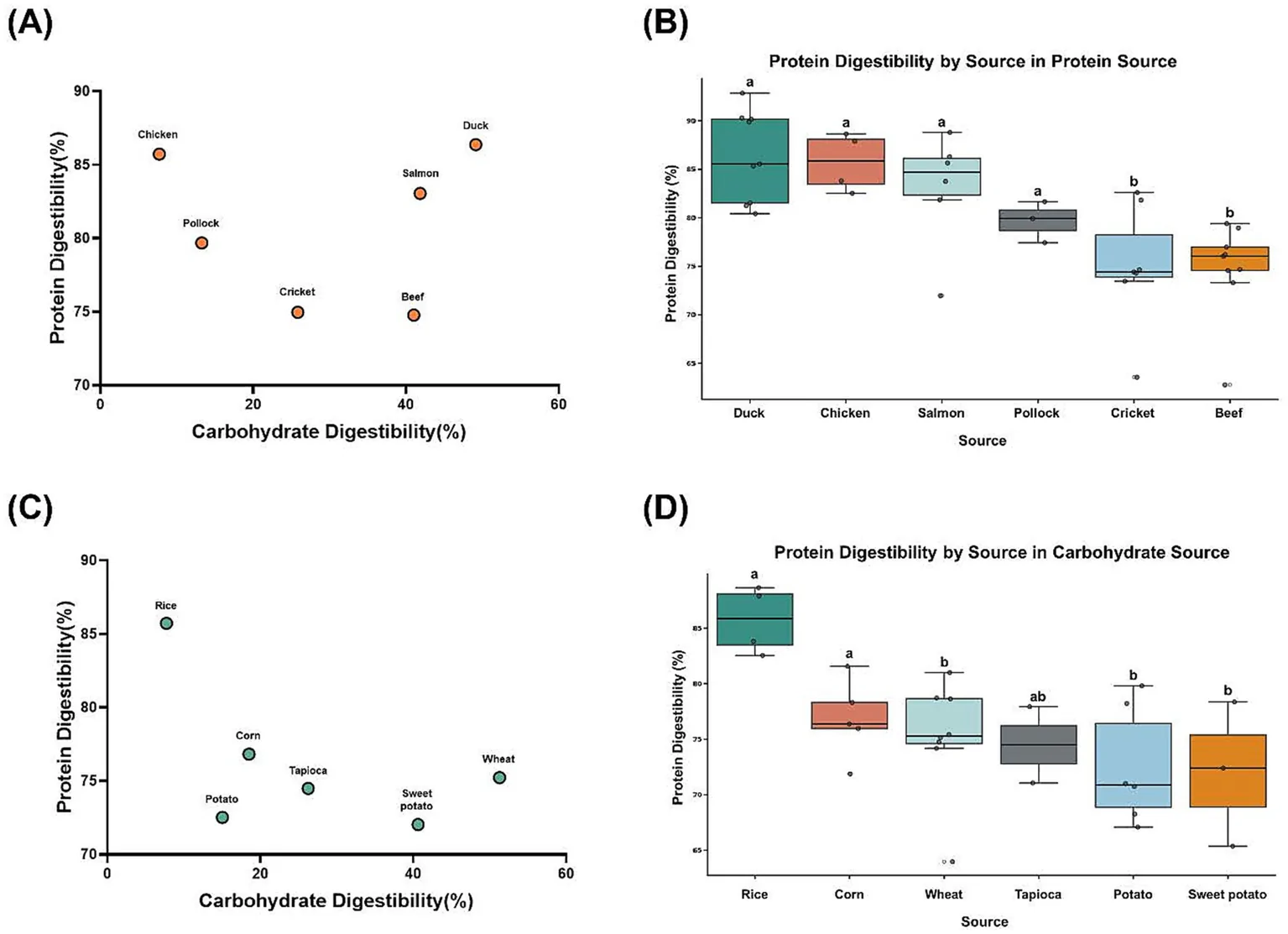
Total tract digestibility of nutrients from distinct dietary sources. **(A)** A scatter plot showing the relationship between protein and carbohydrate digestibility of various single-protein sources. **(B)** A box plot showing protein digestibility of various diverse protein sources. **(C)** A scatter plot showing the relationship between protein and carbohydrate digestibility of various single-carbohydrate sources. **(D)** A box plot showing protein digestibility of various diverse carbohydrate sources. Lowercase letters on top of each box plot represent significant differences according to Tukey’s HSD test, *p* < 0.05. Individual points represent animal-level ATTD observations retained in the analysis.

When digestibility was compared among the single-protein source diets formulated with rice as the reference carbohydrate source, crude protein ATTD differed significantly among dietary protein sources. Duck showed the highest crude protein ATTD (86.36 ± 4.63%), followed by chicken (85.72 ± 3.01%) and salmon (83.05 ± 5.92%). These values were significantly higher than those observed for pollock (79.68 ± 2.12%), cricket (74.97 ± 6.31%), and beef (74.77 ± 4.92%; Figures 2A,B). In contrast, carbohydrate ATTD showed greater within-treatment variability across the protein-source diets, with values of 7.71 ± 13.45% for chicken, 49.15 ± 31.66% for duck, 41.85 ± 38.83% for salmon, 13.27 ± 8.74% for pollock, 41.03 ± 19.54% for beef, and 25.85 ± 20.22% for cricket. These results indicate that crude protein ATTD varied among the tested protein-source formulations within the rice-based dietary matrix, whereas carbohydrate ATTD was more variable among individual cats and was not the primary distinguishing outcome in this evaluation.

### 3.3 Nutrient digestibility in the carbohydrate-source evaluation

When digestibility was compared among the single-carbohydrate source diets formulated with chicken as the reference protein source, carbohydrate ATTD differed among dietary carbohydrate-source formulations. Wheat showed the highest carbohydrate ATTD (51.30 ± 12.30%), followed by sweet potato (40.64 ± 21.25%), tapioca (26.25 ± 6.96%), corn (18.52 ± 9.79%), potato (15.02 ± 12.50%), and rice (7.71 ± 13.45%; Figure 2C). Crude protein ATTD also differed among the carbohydrate-source diets. Rice showed the highest crude protein ATTD (85.72 ± 3.01%), followed by corn (76.82 ± 3.54%), wheat (75.23 ± 5.14%), tapioca (74.49 ± 4.86%), potato (72.52 ± 5.27%), and sweet potato (72.04 ± 6.50%; Figures 2C,D). Rice and corn showed significantly higher crude protein ATTD than the other carbohydrate-source formulations (*p* < 0.05). Because chicken was used as the reference protein source in this evaluation, the lower crude protein ATTD observed in the potato-, sweet potato-, tapioca-, and wheat-containing diets should be interpreted as matrix-specific associations within these fixed formulations rather than as evidence of intrinsic inhibitory effects of the carbohydrate ingredients alone. These results suggest that crude protein ATTD may vary according to the dietary carbohydrate matrix under the tested formulation conditions.

### 3.4 Amino acid ATTD in the protein-source evaluation

**Figure 3:**
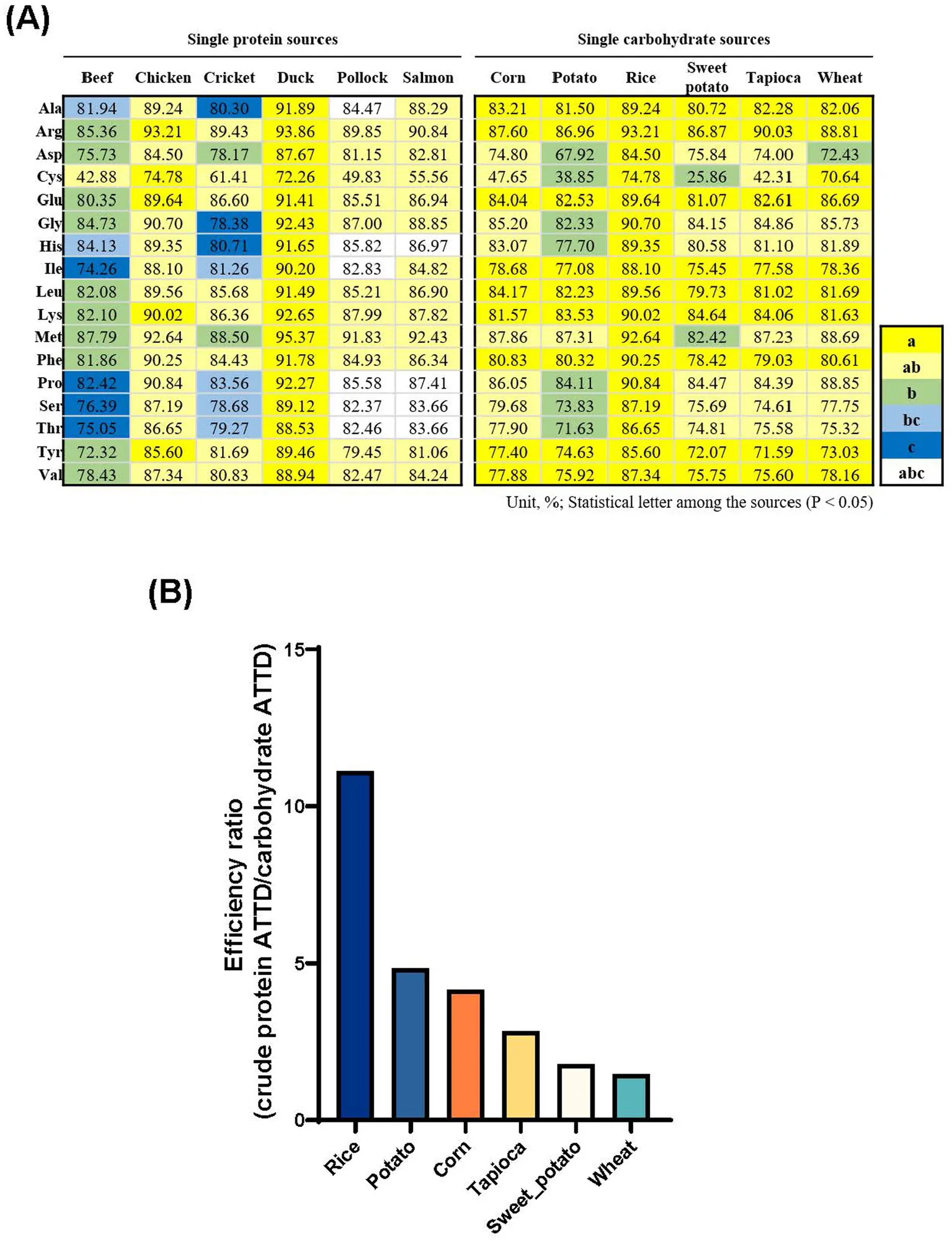
Assessment of amino acid digestibility and the efficiency ratio (ER). **(A)** Assessment of apparent total tract digestibility of amino acids in diets containing different single sources of proteins and carbohydrates. Heatmap values represent treatment means, and the corresponding source-specific mean ± standard deviation values are provided in Sections 3.4 and 3.5. **(B)** Exploratory ER values calculated as the ratio of crude protein digestibility to the carbohydrate digestibility denominator across diets containing different carbohydrate sources. ER values should be interpreted as descriptive protein-to-carbohydrate digestibility ratios rather than direct measures of protein utilization.

When amino acid ATTD was compared among the protein-source diets, duck generally showed high digestibility across the assessed amino acids, followed by chicken. In the duck-source formulation, amino acid ATTD values were high for several representative amino acids, including Ala (91.89 ± 4.19%), Val (88.94 ± 5.88%), Ile (90.20 ± 5.01%), Phe (91.78 ± 5.36%), and Met (95.37 ± 2.88%). Chicken also showed relatively high amino acid ATTD, including Ala (89.24 ± 2.57%), Val (87.34 ± 2.91%), Ile (88.10 ± 3.20%), Phe (90.25 ± 2.67%), and Met (92.64 ± 1.99%). In contrast, beef and cricket showed lower ATTD values for a wider range of amino acids. Beef showed relatively low ATTD for Ile (74.26 ± 6.84%), Thr (75.05 ± 5.06%), Ser (76.39 ± 4.73%), and Pro (82.42 ± 3.46%), whereas cricket showed relatively low ATTD for Ala (80.30 ± 5.36%), Gly (78.38 ± 5.86%), and His (80.71 ± 5.53%; Figure 3A). Cys showed comparatively high variability across several protein-source formulations, including salmon (55.56 ± 27.79%), pollock (49.83 ± 17.85%), beef (42.88 ± 15.30%), and cricket (61.41 ± 13.20%; Figure 3A). These amino acid digestibility patterns indicate that the tested protein-source formulations differed in amino acid ATTD within the rice-based dietary matrix.

### 3.5 Amino acid ATTD in the carbohydrate-source evaluation

Within the carbohydrate-source diets, rice showed the highest overall amino acid ATTD, followed by corn and tapioca. In the rice-source formulation, amino acid ATTD values were consistently high for several representative amino acids, including Ala (89.24 ± 2.57%), Val (87.34 ± 2.91%), Ile (88.10 ± 3.20%), Phe (90.25 ± 2.67%), His (89.35 ± 2.59%), and Met (92.64 ± 1.99%). Corn showed intermediate amino acid ATTD values, including Ala (83.21 ± 3.05%), Val (77.88 ± 4.32%), Ile (78.68 ± 4.08%), Phe (80.83 ± 4.16%), His (83.07 ± 2.41%), and Met (87.86 ± 2.69%). In contrast, potato and sweet potato showed lower ATTD values for several amino acids than the other carbohydrate-source formulations. Potato showed the lowest ATTD for Asp (67.92 ± 7.25%), Gly (82.33 ± 3.76%), His (77.70 ± 5.83%), Pro (84.11 ± 3.62%), Ser (73.83 ± 5.91%), and Thr (71.63 ± 6.33%). Sweet potato showed the lowest ATTD for Cys (25.86 ± 22.39%) and Met (82.42 ± 7.17%; Figure 3A). These amino acid patterns should be interpreted within the specific protein-carbohydrate combinations and processing conditions evaluated in this study.

### 3.6 Exploratory assessment of the efficiency ratio across carbohydrate sources

The results above showed that crude protein ATTD varied across diets containing different carbohydrate sources. To further describe the relative relationship between crude protein ATTD and carbohydrate ATTD, ER was calculated as the ratio of crude protein digestibility to carbohydrate digestibility. Rice showed the highest ER of 11.12, followed by potato (4.82), corn (4.15), tapioca (2.84), sweet potato (1.77), and wheat (1.47; Figure 3B). Thus, wheat and sweet potato showed the lowest ER values among the tested carbohydrate-source formulations. Because ER is calculated as a ratio derived from two percentage-based ATTD values, its magnitude is strongly affected by the carbohydrate ATTD denominator. Therefore, the high ER observed for rice should be interpreted as high crude protein ATTD relative to low carbohydrate ATTD in this formulation, not as direct evidence of increased protein utilization or absorption. These results suggest that ER may provide descriptive information on matrix-dependent digestibility patterns across carbohydrate-source diets, but its biological interpretation remains preliminary.

### 3.7 Candidate indicator amino acids reflecting protein and amino acid ATTD patterns

The coefficient of determination (*R*^2^) analysis was performed to evaluate how closely individual amino acid ATTD paralleled crude protein ATTD across source-level treatment means within each evaluation context. Based on the *post hoc* exploratory threshold of *R*^2^ > 0.82, seven amino acids (Ala, Ser, Val, Thr, His, Phe, and Ile) consistently exceeded the threshold across both protein- and carbohydrate-source groups and were therefore identified as candidate indicator amino acids. Ala showed the strongest linear agreement in both groups, with *R*^2^ values of 0.957 and 0.9831 for the protein- and carbohydrate-source groups, respectively. Val had values of 0.931 and 0.9502, respectively. Within the protein-source group, Ala, Ser (0.9537), and Val showed strong agreement with crude protein ATTD. In the carbohydrate-source group, the strongest agreement was observed for Ala, Ile (0.9738), and Val (Table 4). A comparison between the source groups indicated differences in the strength of these associations. Specifically, His, Phe, and Ile exhibited lower *R*^2^ values in the protein-source group than in the carbohydrate-source group, whereas Ser showed a relatively stronger association within the protein-source group. Because only specific protein-carbohydrate combinations were evaluated, these associations may partly reflect formulation-specific matrix interactions and processing-related effects rather than intrinsic digestibility characteristics of the individual amino acids themselves. Therefore, these amino acids are described as candidate indicators of ATTD patterns rather than validated biomarkers of amino acid bioavailability.

**Table 4:** Coefficients of determination for protein and amino acid digestibility based on protein and carbohydrate sources.

Amino acids (AAs)	Protein-source group (rice-based) *R*^2^ (vs. Cpro)	Carbohydrate-source group (chicken-based) *R*^2^ (vs. Cpro)
Ala	0.957*	0.9831*
Ser	0.9537*	0.8245*
Pro	0.9476*	0.7274
Val	0.931*	0.9502*
Asp	0.9271*	0.733
Thr	0.9103*	0.9282*
Met	0.8956*	0.7341
His	0.8723*	0.927*
Phe	0.8617*	0.9284*
Ile	0.8388*	0.9738*
Gly	0.8171	0.7989
Arg	0.8127	0.7872
Leu	0.8009	0.9222*
Lys	0.7798	0.541
Glu	0.6925	0.7978
Tyr	0.6474	0.878*
Cys	0.5436	0.598

*R*^2^, coefficient of determination; Cpro, crude protein ATTD. *Indicates amino acids exceeding the exploratory threshold of *R*^2^ > 0.82. This threshold was used for candidate indicator selection and was not used as a criterion of statistical significance.

### 3.8 Exploratory association between ER and candidate indicator amino acid ATTD

**Figure 4:**
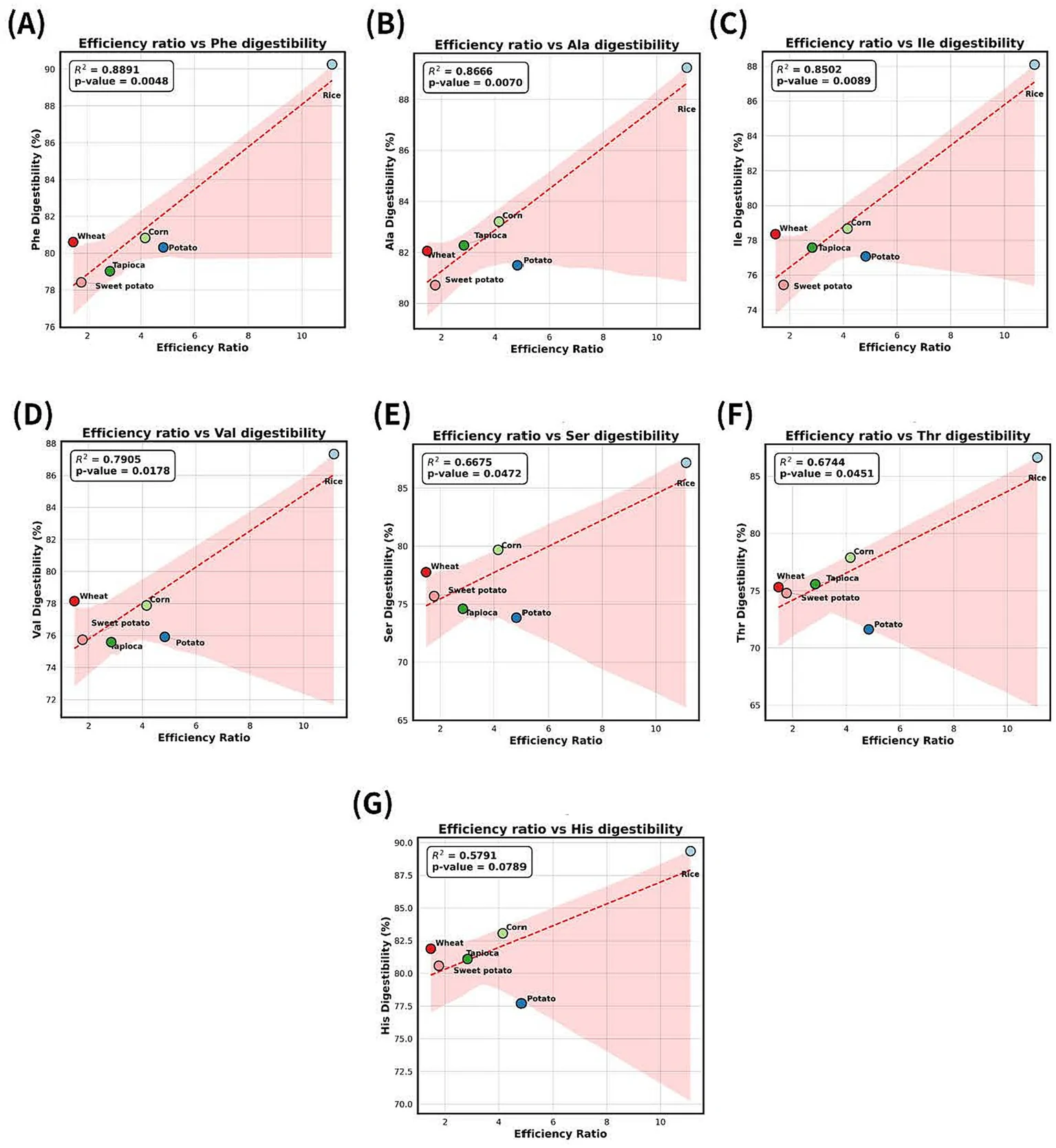
Exploratory linear regression analysis between ER and candidate indicator amino acid ATTD. The scatter plots show group-level associations between ER and the apparent total tract digestibility of selected candidate indicator amino acids across diets containing different carbohydrate sources. Each point represents a dietary treatment mean. Linear regression models show the associations between ER and **(A)** phenylalanine (Phe), **(B)** alanine (Ala), **(C)** isoleucine (Ile), **(D)** valine (Val), **(E)** serine (Ser), **(F)** threonine (Thr), and **(G)** histidine (His). The shaded area represents the 95% confidence band of the fitted regression line. The coefficient of determination (*R*^2^) and *p*-values were calculated for each exploratory regression model. These analyses should not be interpreted as validation of ER as a determinant of amino acid bioavailability or protein utilization.

To explore whether ER was associated with candidate indicator amino acid ATTD across carbohydrate-source diets, simple linear regression analysis was performed between ER and digestibility of the seven candidate amino acids. Six candidate amino acids (Phe, Ala, Ile, Val, Ser, and Thr) showed positive group-level associations with ER (Figures 4A–F), with the strongest relationships observed for Phe, Ala, and Ile. His showed a weaker and non-significant association with ER (*R*^2^ = 0.58, *p* = 0.079; Figure 4G). The scatter plots showed that rice, which had the highest ER (11.12), also had high ATTD values for the candidate amino acids and appeared in the upper-right region of the plots. Because each point represents a dietary treatment mean and the number of carbohydrate-source formulations was limited, these regressions should be interpreted as exploratory group-level associations rather than as validation of ER. The results indicate that ER was associated with selected amino acid ATTD patterns within the tested carbohydrate-source formulations.

## 4 Discussion

For domestic cats, a complete commercial diet is the primary source of daily nutrients. This highlights the need for formulation strategies to be guided not only by nutrient concentration but also by ingredient-specific digestibility and diet-level matrix effects (18, 21). The food matrix can influence the enzymatic accessibility of macromolecules through its structure and physicochemical properties, which may regulate the digestion of proteins and carbohydrates (22–24). Despite this, controlled evidence on how specific single-ingredient matrices affect macronutrient digestibility in domestic cats remains limited. This question is particularly relevant because cats are obligate carnivores that evolved consuming protein-rich diets low in carbohydrates (25).

In domestic cats, dietary protein serves both as a source of amino acids for tissue maintenance and as a major substrate for endogenous glucose production via continuous gluconeogenesis. This reliance on protein is compounded by the limited capacity of cats to downregulate amino acid oxidation during periods of reduced protein intake, resulting in obligatory nitrogen losses and poor metabolic adaptation to low-protein diets (6, 25). Together, these physiological characteristics indicate that feline diet formulation should extend beyond meeting minimum crude protein requirements and should also consider the digestibility of protein and amino acids within the complete diet matrix.

Within this context, the present study examined whether protein-source and carbohydrate-source formulations were associated with variation in apparent total tract digestibility (ATTD) of crude protein and amino acids. Rice was used as the reference carbohydrate source in the protein-source evaluation based on previous feline starch-source studies evaluating rice or brewers rice in extruded cat diets (17, 19). Chicken was used as the reference protein source in the carbohydrate-source evaluation based on previous feline carbohydrate-source work using chicken powder as a constant protein ingredient and prior evaluation of chicken-based protein ingredients intended for dog and cat foods (19, 20). This approach reduced formulation complexity, but it did not constitute a full factorial protein × carbohydrate design. Therefore, the observed responses should be interpreted as matrix-specific outcomes within the tested formulations rather than universal intrinsic effects of each isolated ingredient.

Among the protein-source diets formulated with rice as the reference carbohydrate source, duck, chicken, and salmon showed higher crude protein ATTD than beef and cricket. These differences are consistent with previous pet-food ingredient studies showing that protein sources can differ in chemical composition, amino acid profile, processing conditions, and true nutrient or amino acid digestibility (26). However, because these comparisons were made only within rice-based formulations, the results should be interpreted as responses within this specific matrix context rather than definitive rankings of intrinsic protein-source digestibility across all possible carbohydrate matrices.

The influence of carbohydrate ingredients on apparent protein digestion can be interpreted through the concept of matrix accessibility, whereby the carbohydrate fraction may define the physical and chemical environment governing enzyme diffusion and substrate exposure. Structural features such as plant cell wall integrity, fiber content, particle disintegration, and digesta viscosity can constrain enzyme-substrate interactions and may limit proteolysis when proteins are embedded within dense or viscous carbohydrate matrices (22–24). In the present study, rice- and corn-containing carbohydrate-source diets were associated with higher crude protein ATTD than several other carbohydrate-source formulations. Previous controlled feeding studies in cats have also shown that starch source can influence total tract digestibility, postprandial responses, and gut microbial outcomes (17, 19). Thus, the present results support the relevance of carbohydrate-source selection in extruded cat diets, while the proposed mechanisms related to viscosity, physical obstruction, and enzymatic accessibility should be regarded as plausible explanations rather than directly measured mechanisms.

Because protein digestibility may be influenced by both the protein ingredient and the accompanying carbohydrate matrix, crude protein concentration alone provides an incomplete description of diet-level protein value. Crude protein reflects total nitrogen content but does not account for differences in amino acid release, apparent digestion, or potential ingredient interactions. This limitation parallels broader critiques of protein-quality approaches that rely only on crude protein concentration and supports the value of considering digestible amino acid patterns when evaluating ingredients for companion animal diets (21).

In this study, ER was retained as a descriptive index to summarize the relative relationship between crude protein ATTD and crude fiber ATTD across carbohydrate-source diets. Although ER should not be interpreted as a direct measure of protein utilization, protein absorption, or post-absorptive amino acid bioavailability, it provided a concise way to describe how crude protein ATTD varied relative to crude fiber ATTD within the tested fixed-matrix formulations. The high ER observed for rice reflected high crude protein ATTD relative to the low crude fiber ATTD denominator in that formulation. Since ER is calculated from two percentage-based digestibility values, it is mathematically sensitive to the crude fiber ATTD denominator, and a low crude fiber ATTD value can inflate ER without necessarily indicating a proportional improvement in amino acid absorption. The exploratory regressions between ER and candidate indicator amino acid ATTD further suggested that selected amino acid ATTD patterns covaried with this descriptive ratio across the carbohydrate-source formulations. However, these analyses were based on a limited number of treatment means, and rice, which had the highest ER, appeared as a potential high-leverage formulation in several regression plots. In addition, ER and amino acid ATTD are both derived from digestibility-related measurements and may therefore not be fully independent. Accordingly, these associations should be interpreted as preliminary formulation-level patterns that help describe diet-matrix behavior, rather than as evidence that ER predicts or determines protein bioavailability, amino acid absorption, or post-absorptive utilization.

The candidate indicator amino acids identified in this study showed strong linear agreement with crude protein ATTD across both source categories. These amino acids may provide descriptive markers of amino acid ATTD patterns within the tested formulations. However, the observed associations may partly reflect formulation-specific matrix interactions and processing-related effects rather than intrinsic digestibility characteristics of the amino acids themselves. In addition, fecal ATTD does not distinguish pre-cecal amino acid digestion from hindgut microbial effects and does not provide standardized ileal digestibility or true bioavailability estimates. Therefore, these amino acids should be considered candidate indicators of ATTD patterns, not validated biomarkers of amino acid bioavailability.

Several limitations should be acknowledged. First, the study used fixed reference ingredients rather than a full factorial protein × carbohydrate design. Therefore, potential protein × carbohydrate interaction effects could not be statistically tested or unfolded across all ingredient combinations. In addition, all individual animal-level ATTD observations retained after the adaptation-based eligibility screening were included in the analysis without additional outlier-based exclusion or adjustment; visually extreme observations in the box plots should therefore be interpreted as part of the observed individual variability. Second, as discussed above, ER should be interpreted only as an exploratory descriptive ratio, and ER-related associations should not be considered predictive or mechanistic evidence. Third, the study was based on fecal ATTD and did not apply ileal digestibility, standardized amino acid digestibility, or post-absorptive utilization approaches. Fourth, extrusion and processing-related parameters, including starch gelatinization, Maillard reaction products, particle structure, and viscosity, were not directly measured.

The use of shelter cats also introduced variation in age, body weight, sex distribution, and feed acceptance, and the cohort included some individuals younger than 12 months. These variables were not included as covariates in the primary ANOVA model, and an *a priori* power analysis was not conducted. In addition, feeding cats at a fixed rate of 15 g/kg body weight/day did not fully account for individual maintenance energy requirements related to metabolic body weight, age, activity level, or neuter status. Although daily refusals were measured and actual intake was used for ATTD calculations, these design features limit causal inference and should be considered when interpreting the results. Cat digestibility and palatability protocol studies also indicate that cat-related factors and food-intake variation can influence digestibility and feed preference estimates (27).

Practically, these findings suggest that carbohydrate ingredients may not be fully interchangeable components in feline dry-food formulation under the tested conditions. In the present fixed-matrix formulations, carbohydrate-source diets were associated with variation in apparent crude protein and amino acid total tract digestibility, which may be relevant when cereal grains are replaced with alternative starch sources or when novel protein ingredients are incorporated. Importantly, this study was conducted in a real-world shelter environment rather than in a highly controlled laboratory colony. This setting inevitably introduced heterogeneity in age, body condition, previous nutritional background, sex distribution, feed acceptance, and individual feeding responses, and these factors should be considered when interpreting the results. However, such heterogeneity also reflects the variability commonly encountered among companion cats living in practical environments. Therefore, although the findings should be interpreted as formulation-specific ATTD outcomes rather than as direct evidence of intrinsic ingredient effects, protein bioavailability, or post-absorptive amino acid utilization, the present study provides exploratory field-based evidence that carbohydrate-source selection may be associated with crude protein and amino acid ATTD within defined feline diet formulations. ER and candidate indicator amino acids may provide complementary descriptive information at the diet level; however, their use as predictive or mechanistic criteria for ingredient selection requires further validation using controlled cohorts, factorial formulation designs, ileal digestibility-based methods, and direct physicochemical measurements.

## Data Availability

The raw data supporting the conclusions of this article will be made available by the authors, without undue reservation.
